# Dielectric Properties of Co-Doped TiO_2_ with Mg and Nb for Energy Storage Applications

**DOI:** 10.3390/nano15211632

**Published:** 2025-10-26

**Authors:** L. Ferchaud, J. P. F. Carvalho, S. R. Gavinho, F. Amaral, L. I. Toderascu, G. Socol, L. C. Costa, R. Benzerga, S. Soreto Teixeira

**Affiliations:** 1 University of Rennes, IETR-UMR 6164, IUT de Saint-Brieuc, 22004 Saint Brieuc CEDEX, France; loeiz.ferchaud@etudiant.univ-rennes.fr (L.F.); ratiba.benzerga@univ-rennes1.fr (R.B.); 2 i3N and Department of Physics, University of Aveiro, 3810-193 Aveiro, Portugal; jpfc@ua.pt (J.P.F.C.); silviagavinho@ua.pt (S.R.G.); filipe.amaral@estesc.ipc.pt (F.A.); 3 Polytechnic Institute of Coimbra, Coimbra Health School (ESTeSC), 3046-854 Coimbra, Portugal; 4 National Institute for Laser, Plasma and Radiation Physics, Magurele, 077125 Ilfov, Romania; izabela.jinga@inflpr.ro (L.I.T.); gabriel.socol@inflpr.ro (G.S.)

**Keywords:** high permittivity, dielectric properties, impedance spectroscopy, dielectric relaxation

## Abstract

Titanium dioxide is attractive for energy storage due to its abundance, stability, non-toxicity, low cost, and favorable electronic/optical properties. Colossal permittivity (CP) in co-doped TiO_2_ is mainly linked to defect structures rather than intrinsic bulk behavior. This work studies the dielectric properties of TiO_2_ co-doped with niobium and magnesium, synthesized by solid-state reaction. Grain size effects were examined by varying ball milling parameters of (½Mg½Nb)_0.05_Ti_0.95_O_2_ and then were correlated with structure, morphology, and dielectric response. X-ray diffraction (XRD), infrared spectroscopy (FTIR), scanning electron microscopy and energy-dispersive X-ray spectroscopy (SEM-EDS), X-ray photoelectron spectroscopy (XPS), and impedance spectroscopy (IS) (40 Hz–10^6^ Hz, 150–370 K) were employed for structural, morphological, and electrical characterization. XRD confirmed the rutile phase. For co-doped samples, larger grains yielded higher dielectric constants, reaching high permittivity (*ε*′ = 429, *T* = 300 K, *f* = 10 kHz at 500 rpm for 2 h). Lower loss tangent (tan *δ* = 0.11, *T* = 300 K, *f* = 10 kHz at 200 rpm for 2 h) is linked to Mg segregation at grain boundaries. The most conductive sample showed the highest dielectric constant, suggesting an IBLC polarization mechanism driven by grain boundary effects. XPS confirmed Nb and Mg incorporation, with Ti^4+^ dominant and minor Ti^3+^ from oxygen vacancies and surface hydroxylation/defects.

## 1. Introduction

Electronic devices are embedded in our daily lives, and this trend is predicted to increase in the future. Thus, the demand for high-performance requirements for energy storage and conversion components is increasing, including capacitors and therefore also the dielectric materials that compose them. Due to this, a lot of research is undertaken on dielectric ceramics, bringing new materials with unprecedented properties. Among them, titanium dioxide (TiO_2_) has emerged as a promising candidate, being the simple metal oxide with a very high dielectric constant [1,2], but also owing to its abundance, chemical stability, non-toxicity, optical and electronic properties, low cost, and high photocatalytic efficiency. TiO_2_ has been extensively studied for a wide range of technological applications, from dielectric ceramics and energy storage devices to photocatalysis and solar cells, owing to its abundance, stability, and tunable electronic properties [3,4]. Notwithstanding great dielectric properties, bulk titanium dioxide does not reach colossal permittivity (CP), meaning a dielectric constant higher than 1000. Nevertheless, a huge number of studies are emerging on vastly increasing the dielectric properties by the addition of one or more dopants.

Interestingly, recent studies have reported colossal relative permittivity for co-doped TiO_2_ in the form (A, B)_x_ Ti_1−x_O_2_, where A is pentavalent and B is tri- or bivalent cations [1,2,5,6,7,8,9]. The B cation corresponds to the acceptor, giving an electron and reducing Ti^4+^ to Ti^3+^, while the B cation corresponds to the donor. Many dopants have been tested and compared, with niobium and magnesium emerging as the best combination [2]. Indeed, using Nb as the A pentavalent appears to hugely improve the dielectric constant [5,6,7]. Whereas it is reported that Mg tends to reduce the dielectric loss by filling the grain boundary vacancies or even by creating its own phase in the grain boundaries [10].

The origin behind the CP effect In TiO_2_ is still debated, and several models have been proposed, including the internal barrier layer capacitor (IBLC) model [5,8], electron-pinned defect dipoles (EPDD) [7,11], interfacial and electrode polarization effects [9], and microscopic inhomogeneities or polaronic relaxation [12].

Despite there being no universal consensus regarding the dominant mechanism, IBLC is widely recognized. This model assumes the grains to be semiconductors and the grain boundaries as insulators, hence creating a capacitor inside the material, with grain boundaries as the dielectric and grains as electrodes. Recently, EPDD also made a breakthrough by being successful for (Nb^5+^, In^3+^) co-doped TiO_2_ [11,13,14].

However, regarding the mechanisms involved, it is widely accepted that CP is closely linked to defects in grains rather than the bulk material. Understanding the intricate polarization mechanisms associated with these defects is critical for advancing CP materials, particularly in co-doped TiO_2_.

In the present work, we focused on TiO_2_ co-doped with niobium and magnesium, prepared using the solid-state reaction method, to evaluate the influence of grain size on the dielectric properties by varying the ball milling parameters.

Structural analysis confirmed a pure rutile phase structure, with larger grain sizes associated with a higher dielectric constant, achieving a high permittivity of *ε*′ = 429 (at *T* = 300 K and *f* = 10 kHz) with dopant concentration *x* = 0.05, processed in a ball milling setup at 500 rpm for 2 h. Smaller grains, linked to Mg accumulation at grain boundaries, give a low loss tangent (tan *δ* = 0.11 at *T* = 300 K and *f* = 10 kHz).

## 2. Materials and Methods

**Figure 1:**
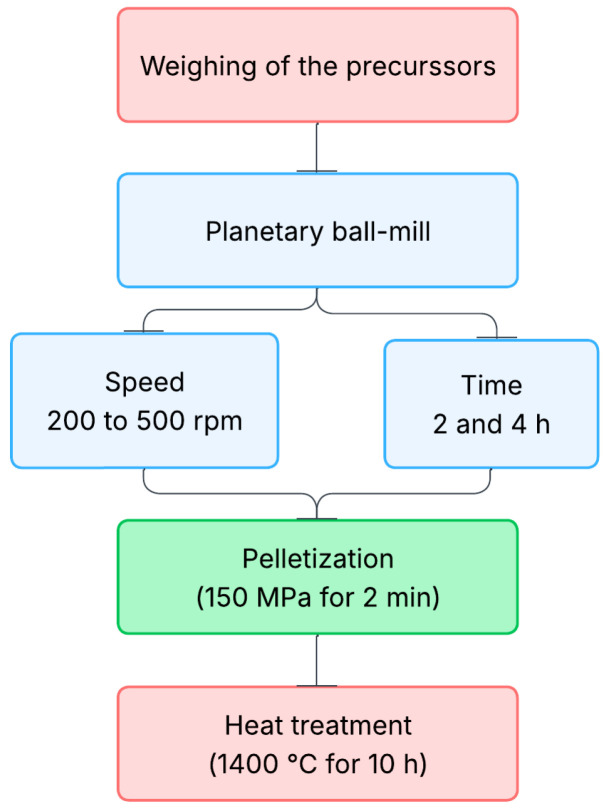
Synthesis route flowchart of the TiO_2_ co-doped samples using different speeds and times of ball-milling.

(Nb+Mg) were prepared by the conventional solid-state reaction, using as raw material TiO_2_ (Alfa Aesar, rutile, 99.5%), MgO (Sigma-Aldrich, 99%), and Nb_2_O_5_ (Sigma-Aldrich, 99.5%) according to the following proportions (½Mg½Nb)_x_ Ti_1−x_O_2_, being x = 0.05. The mixture was then ball-milled in a planetary ball-milling machine (Fritsch Pulverisette 7) with 25 balls (10 mm diameter), 15 g of mixture, and 5 mL of ethanol in an agate vessel. The rotation speed ranged from 200 to 500 rpm for both 2 h and 4 h at each speed. The latter samples are summarized in Table 1 below. For the pellet production, a drop of polyvinyl alcohol (PVA) 2% wt. is added to the powder as a binder, then pressed into cylindrical pellets with a 13 mm diameter in a uniaxial press with a pressure of 150 Mpa for 2 min. The PVA will be removed during the sintering. The sintering is realized at 1400 °C for 10 h with a heating rate of 5 °C/min in a platinum crucible. Moreover, powder corresponding to the pellet has been disposed at the bottom of the crucible as sacrificial powder to prevent the pellets from passing through the crucible. The complete synthesis route is summarized in Figure 1.

The crystallographic structure and the phase formation of the samples were studied by X-ray diffraction (XRD). An AERIS X-ray diffractometer from PANalytical was used, using Cu-K1 radiation (λ = 1.54056 Å) at 40 kV and 15 mA, in the 2θ angle range of 10–70° for 30 min.

Fourier Transform Infrared Spectroscopy (FTIR) analysis was performed using a PerkinElmer Spectrum Two spectrometer equipped with a UATR Two (Universal Attenuated Total Reflectance) accessory. The measurements were conducted on powdered samples in transmittance mode, with 20 scans collected per spectrum over the range of 4000 to 400 cm^−1^. The resulting spectra were used to identify characteristic functional groups and molecular vibrations present in the samples.

The surface, bulk morphology, and chemical composition were evaluated by scanning electron microscopy (SEM) (Vega 3-TESCAN) and energy-dispersive spectroscopy (EDS) (Bruker). The samples were coated with carbon before microscopic observation.

The chemical composition of all samples was analyzed by X-ray photoelectron spectroscopy (XPS) using an ESCALAB Xi+ system (Thermo Scientific Surface Analysis, Waltham, MA, USA), using the Avantage Data System, equipped with a multichannel hemispherical analyzer and a dual X-ray source, operated with Al Kα radiation (hν = 1486.2 eV). The samples were mounted on the analysis stage with conductive carbon tape and subsequently outgassed in the pre-chamber at room temperature under a pressure below 2 × 10^−8^ Torr to eliminate chemisorbed water from their surfaces. The C 1s peak at 284.8 eV was used as the internal reference for energy calibration. Quantitative analysis of surface composition and oxidation states was performed by integrating the core-level peaks after Shirley-type background subtraction, applying the appropriate experimental sensitivity factors available in the Avantage software package (version 5.978). Peak assignments and interpretation of chemical states were further supported by reference data from the NIST XPS Database and the XPS Handbook.

For the electrical measurements, in the frequency range from 40 Hz to 1 MHz, pellets in disk form with a thickness of about 1 mm and a diameter of 10 mm were prepared, and their opposite sides were painted with silver conducting paste. During the electrical measurements, the samples were maintained in a helium atmosphere to improve the heat transfer and eliminate the moisture. These measurements were made using a precision impedance analyzer (Agilent 4294A) in the Cp-Rp configuration, in the temperature range of 150–370 K. The real part, *ε*′, and the imaginary part, *ε*″, of the complex permittivity were calculated [15] using Equations (1) and (2):

(1) $$\varepsilon^{\prime }=C_{p}\frac{d}{A\varepsilon_{0}}$$

(2) $$\varepsilon^{\prime \prime }=\frac{d}{\omega R_{p}A\varepsilon_{0}}$$

where *A* and *d* represent the sample area and thickness, respectively, $\varepsilon_{0}$, the free space permittivity, and *ω* the angular frequency. Impedance spectroscopy can distinguish grains and grain boundaries by analyzing their distinct electrical responses, especially in materials like ceramics.

## 3. Results and Discussion

### 3.1. Structural Analysis

**Figure 2:**
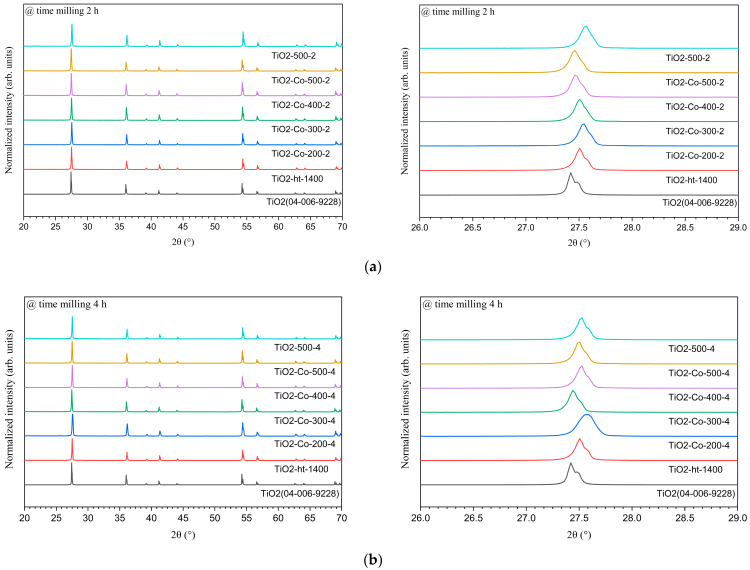
XRD patterns of pure and 5% co-doped TiO_2_ after ball milling for (**a**) 2 h and (**b**) 4 h, compared with pure TiO_2_ heat-treated at 1400 °C (no milling) and the reference pattern from ICDD #04-006-9228. For each figure, on the right side, an inspection at 2θ ≈ 28° is shown.

The XRD pattern for pure and 5% co-doped samples, with both 2 h and 4 h milled time, is shown in Figure 2. All the samples describe a pure rutile TiO_2_ (JCPDS 04-006-9228); nonetheless, after 2θ of 50°, the increase in the speed appears to slightly split the peaks to higher values, without real impact on the structure or the phase as described in Table 2. It displays the crystalline parameters obtained after Rietveld refinement, and these results highlight the pure rutile phase for every 4 h sample, with the goodness-of-fit as an indicator of the phase matching between the sample and the theoretical shape. Thus, it can be affirmed that the ball milling speed as well as the time does not affect the rutile phase and that there is no formation of a major phase from the dopant in the material.

A closer inspection of the main rutile (110) reflection located at approximately 2θ ≈ 28° (right side of Figure 2a,b) reveals a slight shift toward lower diffraction angles for the co-doped samples compared to pure TiO_2_. According to Bragg’s law, this shift corresponds to an increase in the interplanar spacing (dₕₖₗ) and is indicative of lattice expansion. This effect can be attributed to the substitution of Ti^4+^ ions (0.605 Å, CN = 6) by larger dopant cations such as Mg^2+^ (0.72 Å), while Nb^5+^ (0.64 Å) contributes to local distortion and defect compensation mechanisms. Similar peak shifts toward lower 2θ values upon co-doping have been reported in rutile TiO_2_ systems by Yang et al. [16] and Ke et al. [14], confirming dopant incorporation into the TiO_2_ lattice rather than the formation of separate crystalline phases. The subtle variation in unit cell volume observed by Rietveld refinement (Table 2) further supports successful substitutional incorporation of Mg and Nb into the rutile structure with minimal disturbance of the tetragonal symmetry.

**Figure 3:**
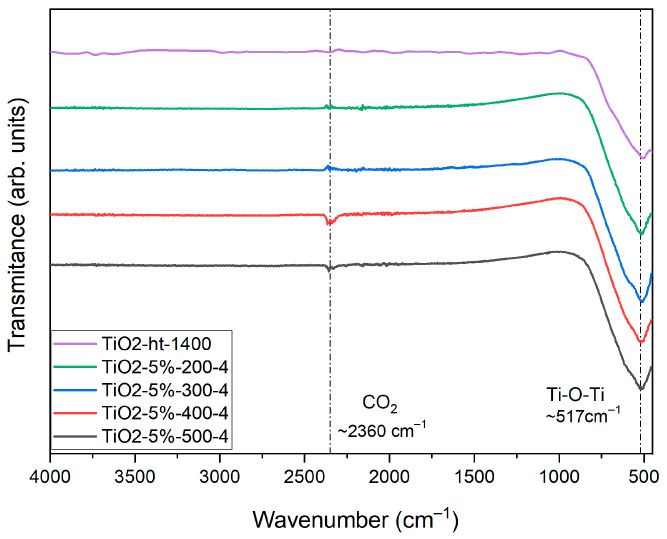
FTIR spectra of pure heat-treated TiO_2_ and co-doped TiO_2_ milled at different speeds for 4 h.

The FTIR spectra of pure heat-treated TiO_2_ and co-doped TiO_2_ samples ground at different rotation speeds, for the longest grinding time, show characteristic vibration bands of Ti–O bonds and adsorbed CO_2_ (Figure 3). The band centered around 517 cm^−1^ is attributed to Ti–O stretching modes, and the weak band near 2360 cm^−1^ corresponds to the symmetric stretching of CO_2_ adsorbed on the surface of the samples. No additional absorption bands indicative of Mg–O or Nb–O bonds were observed in any of the co-doped samples, regardless of the grinding speed. This indicates that even at higher rotation speeds and after prolonged milling, no new vibrational modes involving the dopants were formed that were detectable by FTIR [17,18].

### 3.2. Morphological and Composition Analysis

**Figure 4:**
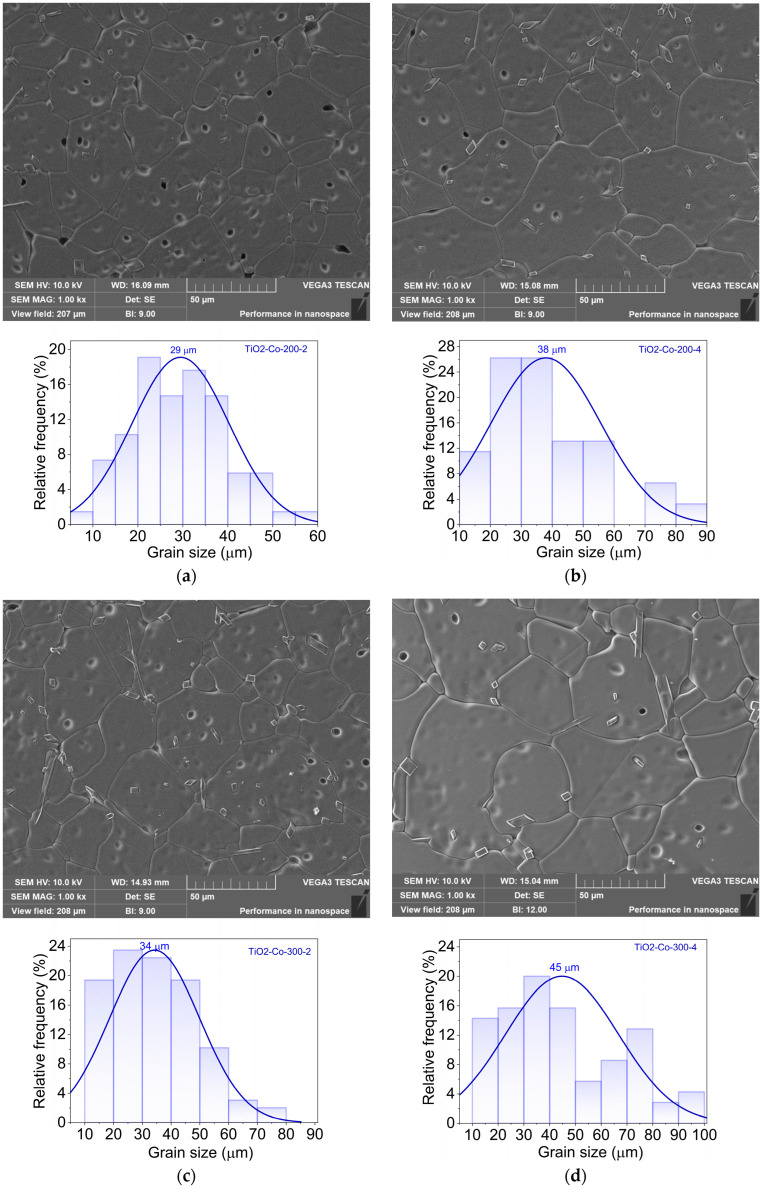

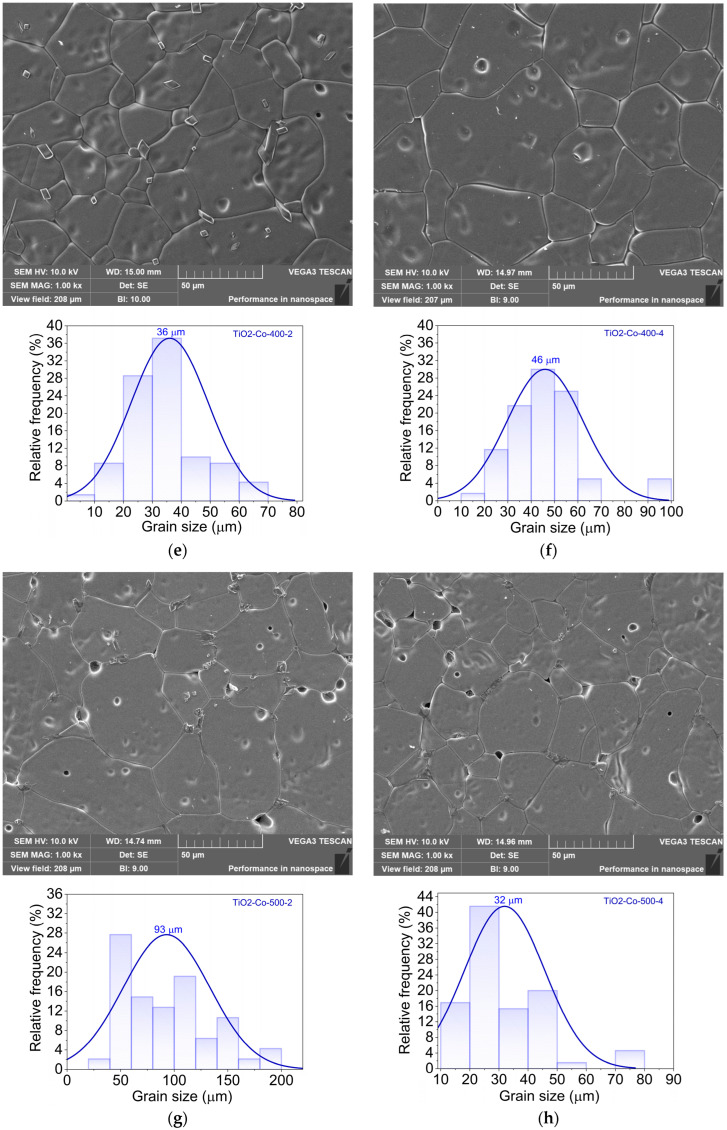
SEM images of all the 5% co-doped samples (magnitude of 10,000×) for (**a**) TiO_2_-Co-200-2, (**b**) TiO_2_-Co-200-4, (**c**) TiO_2_-Co-300-2, (**d**) TiO_2_-Co-300-4, (**e**) TiO_2_-Co-400-2, (**f**) TiO_2_-Co-400-4, (**g**) TiO_2_-Co-500-2, and (**h**) TiO_2_-Co-500-4.

SEM micrographs (Figure 4) reveal dense microstructures for all samples, with grain morphology strongly influenced by both doping and milling conditions.

All samples exhibit a dense microstructure characterized by large TiO_2_ grains, with localized regions of porosity. In general, higher milling speed and longer milling duration promote the formation of ceramic samples with increased grain size. An exception is observed in the sample derived from powders milled at 500 rpm (Figure 4g,h).

Unlike the results of XRD analysis, SEM micrographs point to the existence of localized contrast at some grain boundaries, associated with the presence of an Mg-rich grain boundary phase, presenting a rectangular/rod-like form with less than 2 μm of size. Rather than forming a well-defined crystalline secondary phase, this Mg-rich region is most likely amorphous or nanocrystalline, remaining below the XRD detection limit. This interpretation is supported by EDS mapping, which reveals local enrichment of Mg at grain boundaries, suggesting dopant segregation instead of the formation of a bulk Mg-based oxide phase (e.g., MgO or MgTiO_3_). Similar Mg segregation effects have been reported in co-doped rutile ceramics and are known to contribute to the reduction in dielectric losses by increasing grain boundary resistivity [8,10,16]. The Hume-Rothery rule, which states that a substitutional solid solution is more likely to be formed if the mismatch of the ionic radii is less than 15% and the electronegativities of the two elements are similar, can explain this minor phase [16]. Once the ionic radii of the doping element in the TiO_2_ solution are beyond those limits (52 pm < r < 70 pm), an interstitial solid solution or a secondary phase could be formed. The ionic radii of the Nb^5+^ and Mg^2+^ are 64 pm and 72 pm, respectively. Due to the large ionic radii of the latter, an enriched phase of Mg could be formed. This Mg-rich amorphous or nanocrystalline phase cannot be detected by XRD, probably due to its low volume fraction and/or poor crystallinity, which place its signal below the detection limit of the technique. In addition, its preferential localization at grain boundaries further reduces its contribution to the overall diffraction pattern.

The ball-milling speed and duration also play a critical role. At lower speeds (200 rpm), grains remain relatively small and uniform, whereas increasing the milling speed progressively enlarges the grain size, reaching maximum values at 500 rpm. Interestingly, for 500 rpm, the difference between 2 h and 4 h milling is less pronounced, suggesting a saturation effect in particle refinement and subsequent grain growth during sintering. In contrast, at the lowest and intermediate speeds (200; 300–400 rpm), extending the milling time from 2 h to 4 h noticeably increases the mean grain size by approximately 30%, pointing to enhanced powder homogeneity and reactivity that facilitate grain coalescence during densification.

**Figure 5:**
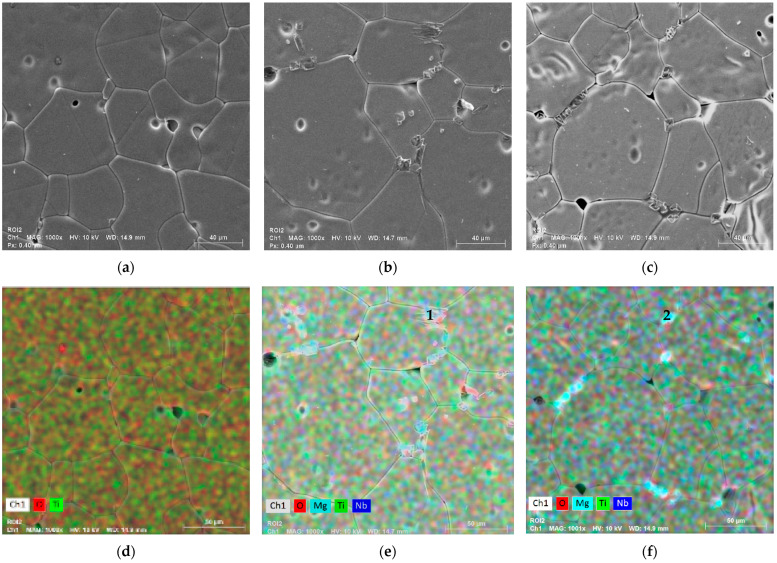
(**a**–**c**) SEM images of undoped and doped samples (magnitude of 10,000×) for (**a**) TiO_2_-500-2, (**b**) TiO_2_-Co-500-2, (**c**) TiO_2_-Co-500-4, and (**d**–**f**) element mapping for samples (**d**) TiO_2_-500-2, (**e**) TiO_2_-Co-500-2, and (**f**) TiO_2_-Co-500-4.

Figure 5 describes the elemental mapping of different samples, allowing us to better understand the distribution of the elements in our samples. Figure 5a exhibits the purity character of the non-doped sample, and Table 3 resumes the homogeneous distribution of titanium and oxygen elements. Figure 5b,c shows the co-doped samples for both 2 h and 4 h, firstly showing the presence of both dopants in the grains. Nevertheless, while niobium tends to increase the grain size [19], it confirms that magnesium creates its own secondary phase in the grain boundaries, subsequently reducing the dielectric loss [10,20]. Thus, Table 3 confirms the reduction in the titanium proportion due to the addition of the dopants. Despite this, the dopant proportions are not evenly distributed, obtaining Mg as a major part. The uneven ratio could be due to this dopant accumulation in the grain boundaries, inducing a higher concentration, raising the Mg reveal on the EDS mapping.

Moreover, as shown in Table 3, the semi-quantitative EDS analysis performed on two additional points (1 and 2) located at grain boundaries revealed a pronounced enrichment in magnesium (≈10–11 at %), confirming Mg segregation in these intergranular regions and supporting the presence of a Mg-rich grain-boundary phase. This result reinforces that no Mg-based crystalline phases were detected by XRD, nor were new Mg–O vibrational bands observed by FTIR, suggesting that the Mg-rich phase is amorphous or nanocrystalline and below the detection limit of conventional diffraction techniques.

**Figure 6:**
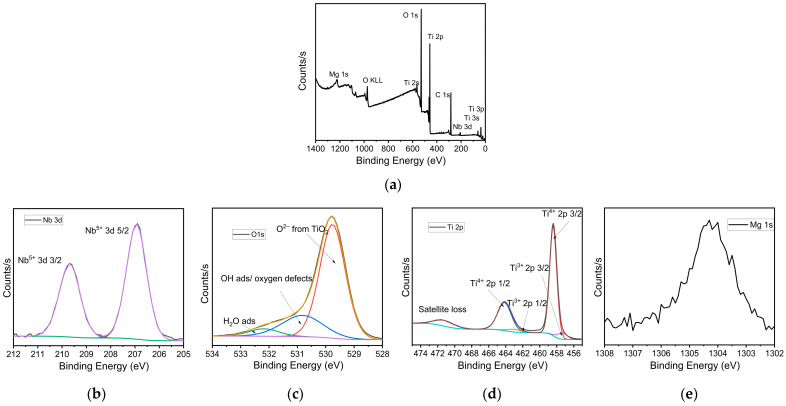
XPS analysis of TiO_2_ co-doped with Nb and Mg: (**a**) survey spectrum and high-resolution spectra of (**b**) Nb 3d, (**c**) O 1s, (**d**) Ti 2p, and (**e**) Mg 1s.

X-ray photoelectron spectroscopy (XPS) was employed to analyze the chemical composition and elemental states present in the doped TiO_2_ pellets. The survey spectrum (Figure 6a) confirms the presence of titanium (Ti), oxygen (O), niobium (Nb), and magnesium (Mg) within the pellet. No significant impurities were detected, indicating a clean synthesis process. High-resolution spectra were acquired for the O 1s, Ti 2p, Nb 3d, and Mg 1s core levels to further investigate the oxidation states and chemical environments of these elements.

The O 1s core-level spectrum (Figure 6c) shows a primary peak centered at approximately 530.0 eV, which is attributed to lattice oxygen (Ti–O) in the TiO_2_ structure. Additional components at higher binding energies (~531.5 eV and ~532.5 eV) are assigned to oxygen vacancies, hydroxyl groups, and possibly adsorbed molecular water or carbonates on the surface [21,22]. The multi-peak deconvolution suggests the coexistence of both stoichiometric TiO_2_ and defect-rich or hydroxylated regions, which can influence the electronic and catalytic properties of the pellet.

In the Ti 2p spectrum (Figure 6d), two distinct peaks are observed at binding energies of ~458.5 eV and ~464.2 eV, corresponding to the Ti 2p_3_/_2_ and Ti 2p_1_/_2_ spin–orbit components, respectively. These positions are consistent with Ti in the +4 oxidation state, confirming the presence of Ti^4+^ as expected in TiO_2_ [23]. Minor shoulders or slight asymmetries in the lower binding energy region hint at the presence of a small fraction of Ti^3+^ species, likely associated with oxygen vacancies or a slight reduction in the titanium environment.

The Nb 3d spectrum (Figure 6a) reveals a well-defined doublet with peaks at approximately 207.5 eV (Nb 3d_5_/_2_) and 210.2 eV (Nb 3d_3_/_2_), characteristic of Nb in the +5 oxidation state [24]. The sharp and symmetrical nature of the peaks suggests that Nb is uniformly incorporated into the TiO_2_ lattice, likely substituting Ti^4+^ sites, which can enhance electrical conductivity and modify the band structure through donor doping.

The Mg 1s core-level peak appears at ~1303 eV, corresponding to Mg^2+^ species [25]. The relatively weak intensity of this peak indicates a lower concentration of magnesium in the pellet, yet its clear presence confirms successful incorporation (Figure 6e). Magnesium may either be substituting for Ti^4+^ in the lattice or present at the surface, potentially influencing the surface charge distribution and photocatalytic behavior of the pellet.

Collectively, the XPS analysis demonstrates that the TiO_2_ film is successfully doped with both Nb and Mg, maintaining the primary Ti^4+^ oxidation state and showing features indicative of oxygen vacancies and surface hydroxylation. These modifications are expected to play a significant role in tuning the pellet’s optical, electronic, or catalytic properties.

### 3.3. Electrical Analysis

Impedance spectroscopy grants us the fundamental aspect of the material properties, notably the molecular motion therein. The complex permittivity has been calculated, ε* = *ε*′ − *iε*”, with both the real part, *ε*′, representing the ability of the material to store energy, and the imaginary part, *ε*″, related to the dissipated energy. Through the complex permittivity components, it is also possible to calculate the dielectric loss, tan *δ*, defined by (Equation (3)),

(3) $$\tan\delta =\frac{\varepsilon^{\prime \prime }}{\varepsilon^{\prime }}$$

**Figure 7:**
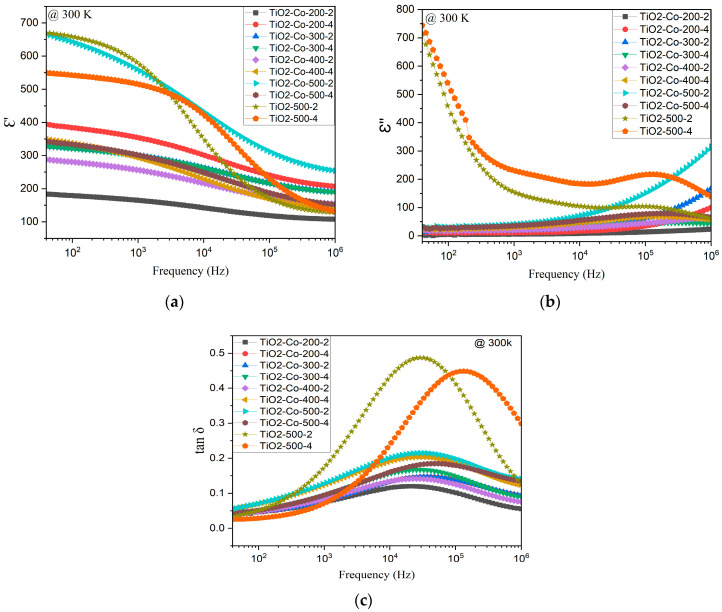
Frequency dependence of the dielectric properties of undoped and 5% doped TiO_2_, (**a**) dielectric constant *ε*′, (**b**) imaginary part *ε*″ at 300 K, and (**c**) loss tangent, tan *δ*.

The frequency dependence (40 Hz to 1 MHz) of the dielectric properties of the 5% co-doped and non-doped rutile TiO_2_ at 300 K is shown in Figure 7. Figure 7a shows the dielectric constant, with all the doped samples appearing to have the same tendency. As observed, samples prepared with higher ball-milling speed present higher dielectric constant values, notably TiO_2_-Co-500-2 reaching the best dielectric constant (e’ = 429) at 10 kHz. At low frequencies, pure TiO_2_ samples display higher values than the co-doped ones. However, in the MHz region, where it is expected that extrinsic polarization mechanisms no longer have a place, the co-doped samples (except the TiO_2_-Co-200-2 sample) present higher e’ than the undoped TiO_2_. This effect can be related to the codoping process, which has a stronger influence on the intrinsic dielectric constant of the samples compared to the impact of the ball-milling conditions.

All the doped samples present the same frequency dependence for the dielectric losses as seen in Figure 7c, and for all co-doped samples, a strong decrease in tan *δ* is observed, compared with the undoped samples. In fact, the dopants affect the imaginary part of Figure 7b by reducing its proportion when compared to the non-doped samples. In addition to this, the ball-milling time also seems to have an impact on the frequency of the relaxation peak; indeed, for the non-doped samples, as can be seen in Figure 7c, 2 h of milling induces a peak at 30 kHz, while 4 h has a peak at 200 kHz. Moreover, the best tan *δ* result was measured for the sample TiO_2_-Co-200-2 with (tan *δ* = 0.11) at 10 kHz. Relating the dielectric response to the sample’s morphology, observed from SEM micrographs, we can state that samples with the lowest dielectric losses correspond to those with the lowest grain size. Based on the previous correlation between the grain size and the composition, it is possible to link the dopant and the dielectric properties. Certainly, niobium may influence the dielectric constant while magnesium influences the dielectric losses, as confirmed in other works on doping with Nb and Mg [5,6,7,10]. Indeed, the strong dielectric dispersion observed at low frequencies (Figure 7a), together with the simultaneous decrease in dielectric loss (tan *δ*) for co-doped samples (Figure 7c), is consistent with an Internal Barrier Layer Capacitance (IBLC) mechanism. This model assumes the coexistence of semiconducting grains and insulating grain boundaries, which leads to charge accumulation at interfaces and results in a Maxwell–Wagner-type relaxation. The improved dielectric response obtained upon co-doping can therefore be understood as a consequence of Nb^5+^ donor doping increasing bulk conductivity, while Mg^2+^ segregation enhances grain boundary resistivity, promoting interfacial polarization. Similar frequency-dependent dielectric behavior driven by IBLC has been widely reported in co-doped TiO_2_ ceramics and other colossal permittivity materials [5,8,26,27]. These results support the conclusion that the dielectric enhancement in the present work is predominantly extrinsic in origin and governed by grain boundary effects rather than intrinsic lattice polarization.

Overall, the dielectric results depend on the ceramics’ microstructure, which in turn depends on the ball-milling conditions. Indeed, the highest dielectric constant is observed for the samples with higher mean grain size, which corresponds to the samples prepared with the highest ball-milling speed (TiO_2_-Co-500-2). On the other hand, the lowest dielectric constant and losses correspond to the sample with the lowest mean grain size, and so the lowest milling speed (TiO_2_-Co-200-2). For context, Table 4 compares our highest *ε*′ and lowest tan *δ* at 10 kHz and 300 K with representative (Mg,Nb)- and (In,Nb)-co-doped rutile TiO_2_ from the literature. While literature colossal permittivity (CP) systems can exceed 10^4^ with very low loss, our work focuses on correlating microstructure (grain size/grain boundary chemistry) with dielectric response under controlled milling and sintering conditions.

**Figure 8:**
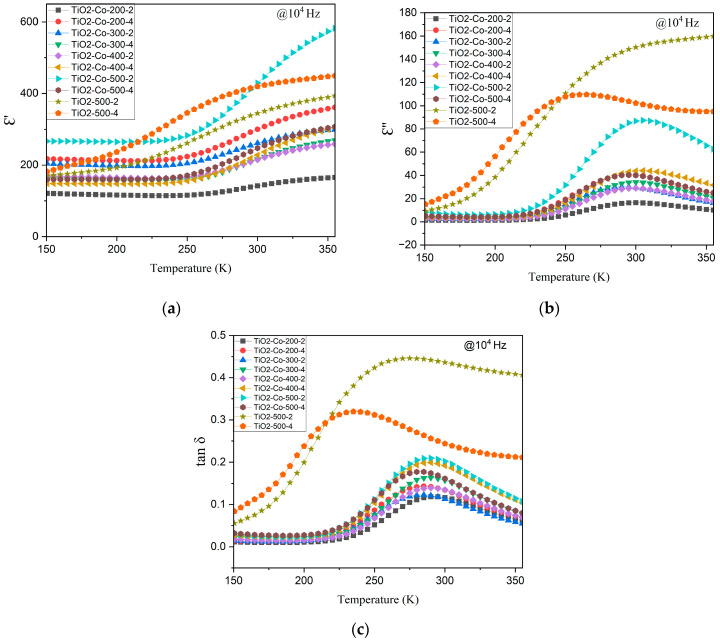
Temperature dependence of the dielectric properties of undoped and doped TiO_2_, (**a**) real part *ε*′, (**b**) imaginary part *ε*″, and (**c**) loss tangent, tan *δ*.

Figure 8 shows the temperature dependence of the dielectric properties from 150 K to 370 K at 10 kHz. At low temperatures, the dielectric constant is stable and starts rising significantly from 250 to 275 K for the doped samples. This increase means that at this temperature, there is enough energy to unlock a new polarization mechanism, enabling the increase in the permittivity of the material. This phenomenon is also visible on the imaginary part, Figure 8b, *ε*″, where the peaks are in the same temperature ranges. It is likely that this temperature-dependent polarization mechanism can be ascribed to the IBLC model. With the rising temperature, more electrons/holes are thermally excited in the semiconducting grains, increasing their mobility and conductivity, and consequently increasing charge storage at the grain/grain boundaries interface. Moreover, the ball-milling time also seems to have an impact on the peak temperature, compared to the pure TiO_2_ curves, TiO_2_-500-2 and TiO_2_-500-4. The lowest ball-milling time causes a higher split of the peak in temperature.

**Figure 9:**
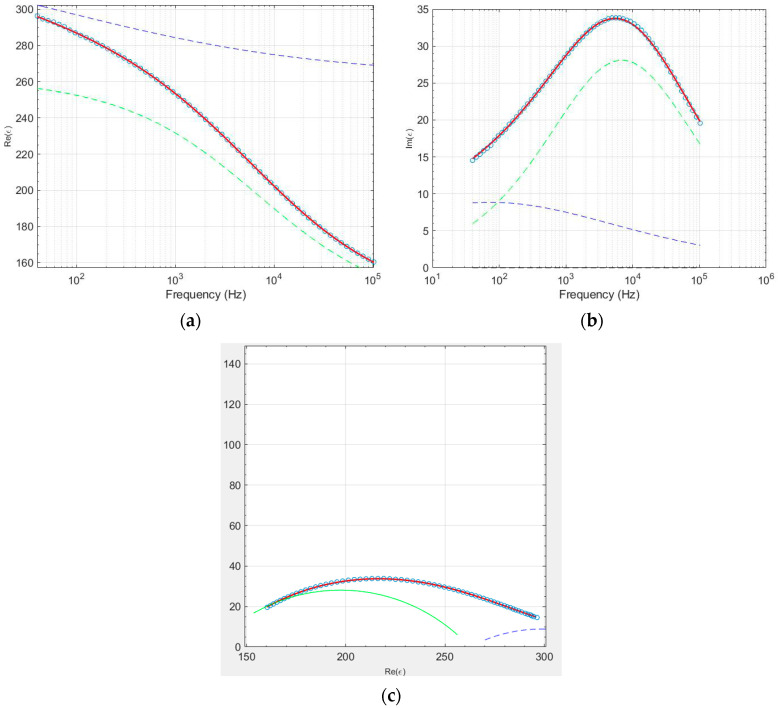
Sample TiO_2_-Co-300-4 at 290 K, (**a**) real part *ε*′ vs. frequency, (**b**) imaginary part *ε*″ vs. frequency, and (**c**) Cole–Cole plot. The red line presents the fitting of the experimental data, where the blue and green dashed lines correspond to each relaxation process.

The information obtained from scanning electron microscopy is important to decide on the best model of the dielectric relaxation to be used. The double relaxation processes are often attributed to the distinct electrical behaviors of the grains (bulk) and grain boundaries. These two microstructural regions contribute separately to the dielectric and conductivity response of the material, resulting in two distinct relaxation processes. Then, we used the Havriliak–Negami model, with two relaxation processes, which fit the data accurately. Figure 9 shows the experimental data and the fitting for the sample TiO_2_-Co-300-4 at 290 K. In the Cole–Cole plot, Figure 9c, the high-frequency arc corresponds to grain response, and the low-frequency arc corresponds to grain boundary response. Similarly, in dielectric permittivity versus frequency plots, a shoulder or peak at high frequencies is attributed to the grain relaxation, and a second shoulder or peak at lower frequencies arises from the grain boundaries.

To obtain the two relaxation parameters, the expression given by S. Havriliak et al. [28] was used (Equation (4)):

(4) $$\varepsilon^{*}\left(\omega \right)\;=\;\frac{\sigma_{dc}}{i\omega \varepsilon_{0}}+\varepsilon_{\infty }+\frac{\Delta \varepsilon_{1}}{{\left(1+{\left(i\omega \tau_{1}\right)}^{\alpha_{1}}\right)}^{\beta_{1}}}+\frac{\Delta \varepsilon_{2}}{{\left(1+{\left(i\omega \tau_{2}\right)}^{\alpha_{2}}\right)}^{\beta_{2}}}$$

where ε*(*ω*) is the complex dielectric permittivity as a function of angular frequency, *ω*, (*ω* = 2π*f*), *σ_dc_* is the conductivity of the material under direct current, *ε*_0_ is the free space permittivity, *ε*_∞_ is the permittivity at high frequencies, ∆*ε* = *ε_S_* − *ε*_∞_ is the dielectric strength, with *ε_S_* being the static permittivity (*ω* = 0), *τ* being the relaxation times, and *α* and *β* being the shape parameters of both relaxations that affect the width and asymmetry of the relaxation process.

**Figure 10:**
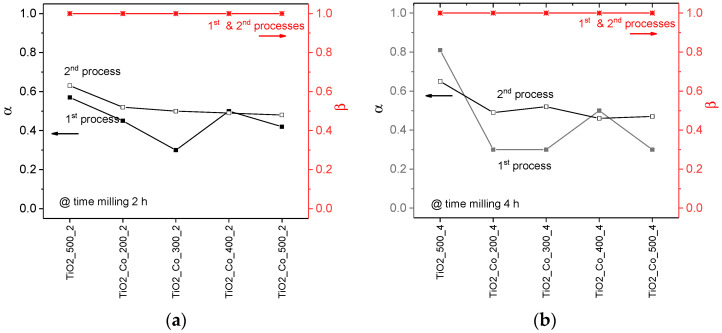
Shape parameters, *α* and *β*, for all pure TiO_2_ and co-doped TiO_2_ samples for a time of milling of (**a**) 2 h and (**b**) 4 h, for relaxation processes 1 and 2.

The HN model was fitted to the experimental data by using the software EIS smart tool [29]. The fitting results are in agreement with the experimental data, meaning that the HN model can be used to accurately describe their behavior. The analysis of the Havriliak–Negami parameters (Figure 10) reveals that only the *α* parameter deviates from unity, while *β* remains equal to one for both the first and second relaxation processes. This behavior corresponds to a non-Debye dielectric response of the Cole–Cole model, indicating a moderate distribution of relaxation times associated with microstructural heterogeneities, such as grain size variations and Mg-rich grain-boundary regions.

According to the HN relaxation formula, if *β* = 1, the relaxation mechanism becomes a Cole–Cole model [28] (Equation (5)):

(5) $$\varepsilon^{*}\left(\omega \right)\;=\;\varepsilon_{\infty }+\frac{\Delta \varepsilon }{1+{\left(i\omega \tau \right)}^{\alpha }}$$

The efficient fitting of the data now allows us to extract *f*_max_ and *τ*_max_. In a manner to obtain the activation energy, the Arrhenius behavior equation can be used (Equation (6)):

(6) $$\tau \;=\;\tau_{0}\;exp\left(-\frac{E_{a}}{K_{B}T}\right)$$

**Figure 11:**
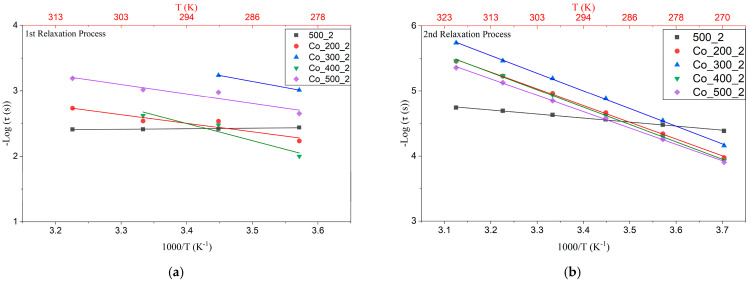
Arrhenius plots of the two relaxations for non-doped samples and 5% co-doped TiO_2_ milled for 2 h: (**a**) 1st relaxation process (lower frequency region) and (**b**) 2nd relaxation process (higher frequency region).

Figure 11 shows the Arrhenius behavior for the 2 h non-doped and 5% co-doped samples for both the 1st (Figure 11a) and 2nd (Figure 11b) relaxation processes. The associated activation energies are summarized in Table 5. As displayed in Figure 11, the co-doped samples exhibit *E_a_* well above the non-doped samples. Pure TiO_2_ 1st and 2nd relaxation process energies could correspond to the polaron relaxations due to hopping motions of trapped electrons and/or holes, according to their low values of activation energy (from 10 to 100 meV), widely studied [12,30,31,32]. While for the co-doped sample, values go closer to one eV activation energy, indicating the displacement of ions such as oxygen vacancies or dipoles in oxides [14]. It is believed to be a Maxwell–Wagner relaxation, due to the importance of the extrinsic dielectric response in CP materials [12].

The spread of activation energy for the first relaxation (~0.26–0.92 eV) among doped samples is attributed to variations in grain-boundary barrier height within an IBLC framework, governed by (i) microstructure (grain size and grain boundary (GB) area/thickness, controlled by milling) and (ii) Mg-rich GB segregation, which increases GB resistivity and the effective barrier. In contrast, the second relaxation exhibits a narrower activation energy range (~0.50–0.57 eV), indicating a less chemistry-sensitive interfacial pathway. The anomalously low value for TiO_2_-Co-200-4 likely reflects partial overlap and limited temperature leverage for the first relaxation and is flagged as low confidence.

**Figure 12:**
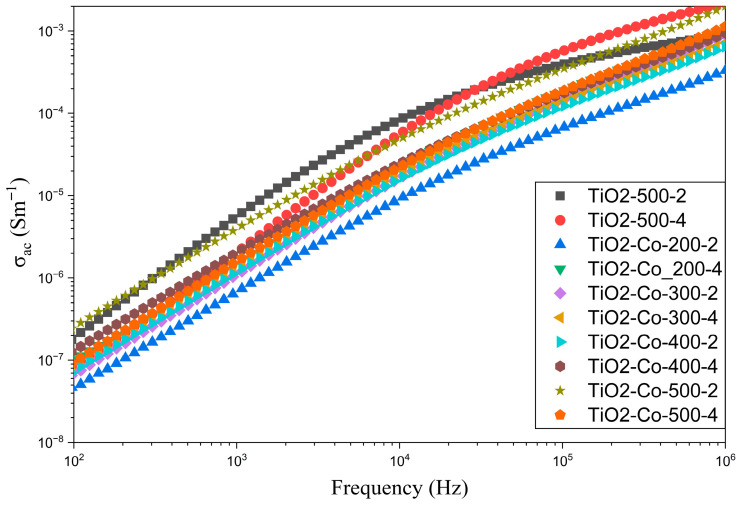
Frequency dependence of the conductivity for non-doped (TiO_2_) and co-doped samples (5% co-doped TiO_2_).

The frequency dependence of the conductivity was also measured to better understand the sample’s properties, as can be seen in Figure 12. The figure shows that the co-doped samples display lower conductivity compared to the non-doped rutile. It can also be seen that the samples milled with higher rpm present higher conductivity for the entire frequency range. Meanwhile, the co-doped sample with the highest conductivity, TiO_2_-Co-500-2, has the highest dielectric constant. This means that the polarization mechanism could be associated with the conductivity difference between grain and grain boundaries, as proposed by the IBLC model.

**Figure 13:**
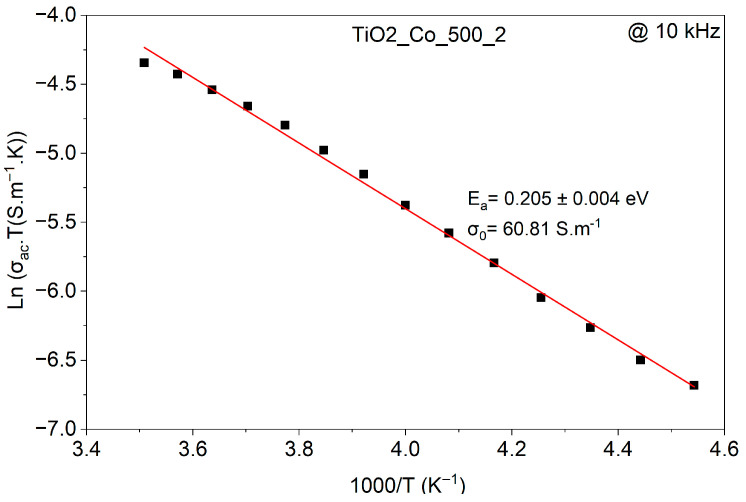
Arrhenius plot of the ac conductivity for TiO_2_-Co-500-2.

The EIS smart tool was also employed to calculate the activation energy using the Arrhenius model. For this purpose, a linear fit was performed by plotting the natural logarithm of the conductivity as a function of 1000/T at 10 kHz, as shown in Figure 13. The temperature dependence of the ac conductivity can be described by an Arrhenius-type behavior:

(7) $$\sigma_{ac}(T)\;=\;\frac{\sigma_{0}}{T}\;exp\left(-\frac{E_{a}}{K_{B}T}\right)$$

where $\sigma_{0}$ is the pre-exponential factor. By plotting $Ln\left(\sigma_{ac}\left(T\right).T\right)$ versus 1000/T, the slope of the linear fit yields the activation energy, $E_{a}$. This formalism is commonly applied in dielectric ceramics to clarify the dominant conduction mechanism [12,25]. The corresponding activation energies are summarized in Table 5.

The samples milled at the highest rpm display the highest dielectric constant and tangent loss, including TiO_2_-Co-500-2, while the lowest rpm induces the lowest values of dielectric constant and tangent loss, particularly TiO_2_-Co-200-2. Nevertheless, a good balance between these two properties is crucial to developing the best material for energy storage. In addition, the grain size was found to be higher at higher speeds, confirming that higher grain size provides higher dielectric constant values. On the other hand, samples with the lowest rpm, and so the smallest grains, obtain the lowest values of tangent loss. As discussed previously, magnesium could also be the origin of the reduction in dielectric losses, creating its own phase in the vicinity of TiO_2_ grain boundaries, as displayed by SEM images. Thus, it has been confirmed that ball-milling has a significant impact on sample morphology and that morphology has a crucial role in the dielectric properties. Despite the high dielectric constant observed for TiO_2_-Co-500-2, its dielectric properties remain significantly lower than those reported in other studies on co-doped rutile titanium dioxide [2,3,5,6,7,9,11,13,15], suggesting that the EPDD polarization mechanism could not be present. Nonetheless, to mitigate the high dielectric loss of the samples, the titanium ceramic powders could be incorporated into a polymer nanocomposite (PCN), thereby enabling the design of a flexible energy storage device (ESD).

Co-doping with 5% has been made in this work, but a deepening of the dopant concentration could also be important, according to the impact of the dopant on morphology and, consequently, the dielectric properties.

## 4. Conclusions

The dielectric properties of non-doped and co-doped rutile titanium dioxide were investigated. The first principal purpose was to influence the grain size using a planetary ball mill. Then, the deep relationship between morphology and dielectric properties has been studied. To further investigate their origin and the mechanism behind it, further analysis has been made on their electrical properties, notably the activation energy. The sample with the highest dielectric constant corresponds to the highest milling speed (TiO_2_-Co-500-2), while the sample with the lowest tangent loss corresponds to the lowest milling speed (TiO_2_-Co-200-2), with, respectively, the largest and smallest grain size. This work demonstrates the crucial role of the dopant and the morphology on the dielectric properties, potentially offering various ways to enhance the electrical properties of materials designed for energy storage applications.

## Figures and Tables

**Table 1:** List of co-doped TiO_2_ samples studied and their various synthesis conditions.

Sample	Composition	Speed (rpm)	Time (h)
TiO_2_-500-2	TiO_2_	500	2
TiO_2_-500-4	TiO_2_	500	4
TiO_2_-Co-200-2	(Nb_½_Mg_½_)_0.05_Ti_0.95_O_2_	200	2
TiO_2_-Co-200-4	(Nb_½_Mg_½_)_0.05_Ti_0.95_O_2_	200	4
TiO_2_-Co-300-2	(Nb_½_Mg_½_)_0.05_Ti_0.95_O_2_	300	2
TiO_2_-Co-300-4	(Nb_½_Mg_½_)_0.05_Ti_0.95_O_2_	300	4
TiO_2_-Co-400-2	(Nb_½_Mg_½_)_0.05_Ti_0.95_O_2_	400	2
TiO_2_-Co-400-4	(Nb_½_Mg_½_)_0.05_Ti_0.95_O_2_	400	4
TiO_2_-Co-500-2	(Nb_½_Mg_½_)_0.05_Ti_0.95_O_2_	500	2
TiO_2_-Co-500-4	(Nb_½_Mg_½_)_0.05_Ti_0.95_O_2_	500	4

**Table 2:** Crystalline parameters obtained with Rietveld refinement on the 4 h, 5% co-doped TiO_2_.

Sample	TiO_2_-500-4	TiO_2_-Co-200-4	TiO_2_-Co-300-4	TiO_2_-Co-400-4	TiO_2_-Co-500-4
Crystalline phase	Rutile TiO_2_	Rutile TiO_2_	Rutile TiO_2_	Rutile TiO_2_	Rutile TiO_2_
Crystalline system	Tetragonal	Tetragonal	Tetragonal	Tetragonal	Tetragonal
Space group	P4_2_/mnm	P4_2_/mnm	P4_2_/mnm	P4_2_/mnm	P4_2_/mnm
Volume (Å^3^)	62.41551	62.64447	62.68064	62.67346	62.65655
Goodness-of-fit	3.29971	2.85369	3.15049	3.06936	3.23643

**Table 3:** EDS results for the samples TiO_2_-500-2, TiO_2_-Co-500-2, and TiO_2_-Co-500-4; points 1 and 2 also include the semi-quantitative analysis of magnesium accumulation in grain boundaries.

Atomic %	Ti	O	Nb	Mg
TiO_2_-500-2	30.22 ± 2.79	69.78 ± 0.89	-	-
TiO_2_-Co-500-2	27.59 ± 2.42	70.82 ± 0.86	0.63 ± 0.13	0.96 ± 0.07
TiO_2_-Co-500-4	27.87 ± 2.19	70.72 ± 0.81	0.66 ± 0.12	0.77 ± 0.05
1	28.54 ± 1.49	60.14 ± 4.71	0.13 ± 0.05	11.19 ± 0.56
2	24.63 ± 1.38	64.55 ± 5.20	0.21 ± 0.06	10.58 ±0.57

**Table 4:** Room-temperature (300 K) dielectric performance at 10 kHz for rutile TiO_2_ (this work vs. literature).

Sample	*ε*′ (10 kHz, 300 K)	tan *δ* (10 kHz, 300 K)	Reference
TiO_2_-Co-500-2	429	0.18	This study
TiO_2_-Co-200-2	180	0.11	This study
(Mg,Nb) co-doped rutile TiO_2_ (Mg_1/3_Nb_2/3_)_x_Ti_(1−x)_O_2_, x = 0.5–7%	~2 × 10^4^–4 × 10^4^	~0.008–0.02	[16]
(In,Nb) co-doped rutile TiO_2_	~1 × 10^4^–1 × 10^5^	<0.05	[14]
(Sn,Ta) co-doped rutile TiO_2_ (Sn_1/2_Ta_1/2_)_x_ ti(_1−x_)O_2_, x = 1–5%	~3.6 × 10^4^–4 × 10^4^	~0.015–0.06	[8]
(La,Nb) co-doped rutile TiO_2_ (La_1/2_Nb_1/2_)_x_ ti(_1−x_)O_2_, x = 2–7%	~3 × 10^4^–6 × 10^4^	~0.3–0.35	[19]
(Co,Nb) co-doped rutile TiO_2_	~7 × 10^3^–2.5 × 10^4^	<0.1	[5]

**Table 5:** Activation energy results for both undoped and 5% doped samples, using the frequency or the conductivity methods.

Samples	1st Process *E_a_* (eV) (*τ*_1_)	2nd Process *E_a_* (eV) (*τ*_2_)	*E_a_* (eV) (*σ_ac_*)
TiO_2_-500-2	0.02 ± 0.01	0.12 ± 0.01	0.115 ± 0.001
TiO_2_-500-4	0.04 ± 0.02	0.12 ± 0.01	0.098 ± 0.001
TiO_2_-Co-200-2	0.26 ± 0.06	0.51 ± 0.01	0.221 ± 0.004
TiO_2_-Co-200-4	0.02 ± 0.01	0.53 ± 0.01	0.205 ± 0.003
TiO_2_-Co-300-2	0.36 ± 0.04	0.54 ± 0.01	0.205 ± 0.003
TiO_2_-Co-300-4	0.92 ± 0.16	0.57 ± 0.01	0.209 ± 0.005
TiO_2_-Co-400-2	0.52 ± 0.15	0.53 ± 0.01	0.211 ± 0.004
TiO_2_-Co-400-4	0.66 ± 0.11	0.55 ± 0.01	0.210 ± 0.003
TiO_2_-Co-500-2	0.29 ± 0.06	0.50 ± 0.01	0.219 ± 0.003
TiO_2_-Co-500-4	0.89 ± 0.13	0.55 ± 0.01	0.200 ± 0.004

## Data Availability

The original contributions presented in this study are included in the article. Further inquiries can be directed to the corresponding author.

## References

[1] Diebold U. (2003). The Surface Science of Titanium Dioxide. Surf. Sci. Rep..

[2] Zhou W., Cao M., Wang H., Hao H., Yao Z., Liu H. (2022). Defect Structure Design of TiO_2_ Ceramics with Colossal Permittivity by Doping with Ti Metal Powder. Ceram. Int..

[3] Suchikova Y., Nazarovets S., Konuhova M., Popov A.I. (2025). Binary Oxide Ceramics (TiO_2_, ZnO, Al_2_O_3_, SiO_2_, CeO_2_, Fe_2_O_3_, and WO_3_) for Solar Cell Applications: A Comparative and Bibliometric Analysis. Ceramics.

[4] Liang X., Yu S., Meng B., Wang X., Yang C., Shi C., Ding J. (2025). Advanced TiO_2_-Based Photoelectrocatalysis: Material Modifications, Charge Dynamics, and Environmental–Energy Applications. Catalysts.

[5] Nachaithong T., Chanlek N., Moontragoon P., Thongbai P. (2021). The Primary Origin of Excellent Dielectric Properties of (Co, Nb) Co-doped Tio_2_ Ceramics: Electron-pinned Defect Dipoles vs. Internal Barrier Layer Capacitor Effect. Molecules.

[6] Zhao L., Wang J., Gai Z., Li J., Liu J., Wang J., Wang C., Wang X. (2019). Annealing Effects on the Structural and Dielectric Properties of (Nb + In) Co-Doped Rutile TiO_2_ Ceramics. RSC Adv..

[7] Tsuji K., Han H.S., Guillemet-Fritsch S., Randall C.A. (2017). Dielectric Relaxation and Localized Electron Hopping in Colossal Dielectric (Nb,In)-Doped TiO_2_ Rutile Nanoceramics. Phys. Chem. Chem. Phys..

[8] Mingmuang Y., Chanlek N., Moontragoon P., Srepusharawoot P., Thongbai P. (2022). Effects of Sn^4+^ and Ta^5+^ Dopant Concentration on Dielectric and Electrical Properties of TiO_2_: Internal Barrier Layer Capacitor Effect. Results Phys..

[9] Li Z., Wu J., Xiao D., Zhu J., Wu W. (2016). Colossal Permittivity in Titanium Dioxide Ceramics Modified by Tantalum and Trivalent Elements. Acta Mater..

[10] Li W., Qiu S., Chen N., Du G. (2010). Enhanced Dielectric Response in Mg-Doped CaCu_3_Ti_4_O_12_ Ceramics. J. Mater. Sci. Technol..

[11] Hu W., Liu Y., Withers R.L., Frankcombe T.J., Norén L., Snashall A., Kitchin M., Smith P., Gong B., Chen H. (2013). Electron-Pinned Defect-Dipoles for High-Performance Colossal Permittivity Materials. Nat. Mater..

[12] Wang C., Zhang N., Li Q., Yu Y., Zhang J., Li Y., Wang H. (2015). Dielectric Relaxations in Rutile TiO_2_. J. Am. Ceram. Soc..

[13] Cheng X., Li Z., Wu J. (2015). Colossal Permittivity in Ceramics of TiO_2_ Co-Doped with Niobium and Trivalent Cation. J. Mater. Chem. A Mater..

[14] Ke S., Li T., Ye M., Lin P., Yuan W., Zeng X., Chen L., Huang H. (2017). Origin of Colossal Dielectric Response in (In + Nb) Co-Doped TiO_2_ Rutile Ceramics: A Potential Electrothermal Material. Sci. Rep..

[15] Mckubre M., Macdonald D., Strømme M. (2018). Impedance Spectroscopy: Theory, Experiment, and Applications.

[16] Yang C., Tse M.Y., Wei X., Hao J. (2017). Colossal Permittivity of (Mg + Nb) Co-Doped TiO_2_ Ceramics with Low Dielectric Loss. J. Mater. Chem. C Mater..

[17] El Mragui A., Logvina Y., da Silva L.P., Zegaoui O., da Silva J.C.E. (2019). Synthesis of Fe-and Co-Doped TiO_2_ with Improved Photocatalytic Activity under Visible Irradiation toward Carbamazepine Degradation. Materials.

[18] Khan M.A.M., Siwach R., Kumar S., Alhazaa A.N. (2019). Role of Fe Doping in Tuning Photocatalytic and Photoelectrochemical Properties of TiO_2_ for Photodegradation of Methylene Blue. Opt. Laser Technol..

[19] Wang X.W., Zheng Y.P., Liang B.K., Zhang G., Shi Y.C., Zhang B.H., Xue L.L., Shang S.Y., Shang J., Yin S.Q. (2020). Preparation and Properties of La and Nb Co-Doped TiO_2_ Colossal Dielectric Ceramic Materials. J. Mater. Sci. Mater. Electron..

[20] Patterson E.A., Kwon S., Huang C.C., Cann D.P. (2005). Effects of ZrO_2_ Additions on the Dielectric Properties of CaCu_3_Ti_4_O_12_. Appl. Phys. Lett..

[21] Yu M., Sun H., Huang X., Yan Y., Zhang W. (2020). In Situ-Formed and Low-Temperature-Deposited Nb:TiO_2_ Compact-Mesoporous Layer for Hysteresis-Less Perovskite Solar Cells with High Performance. Nanoscale Res. Lett..

[22] Xu Y., Wu S., Wan P., Sun J., Hood Z.D. (2017). Introducing Ti3+ Defects Based on Lattice Distortion for Enhanced Visible Light Photoreactivity in TiO_2_ Microspheres. RSC Adv..

[23] Wang Y., Qiu C., Xie Y., Wang L., Ding J., Zhang J., Wan H., Guan G. (2024). Intentionally Introducing Oxygen Vacancies and Ti^3+^ Defects on the Surface of Bi_4_Ti_3_O_12_ Nanosheets for Promoting the Photoreduction of CO_2_ to CH_3_OH. ACS Appl. Nano Mater..

[24] Xu W., Russo P.A., Schultz T., Koch N., Pinna N. (2020). Niobium-Doped Titanium Dioxide with High Dopant Contents for Enhanced Lithium-Ion Storage. ChemElectroChem.

[25] Moulder J.F., Stickle W.F., Sobol P.E., Bomben K.D. (1995). Handbook of X-Ray Photoelectron Spectroscopy.

[26] Tsyganov A., Morozova N., Vikulova M., Asmolova A., Zotov I., Bainyashev A., Gorokhovsky A., Gorshkov N. (2024). Thermal Behavior of La^3+^, Ni^2+^ and Sn^4+^ Co-Doped CaCu_3_Ti_4_O_12_ Ceramics Dielectric Response. Inorg. Chem. Commun..

[27] Tsyganov A., Morozova N., Vikulova M., Asmolova A., Artyukhov D., Zotov I., Gorokhovsky A., Gorshkov N. (2023). The Effect of Lithium Doping on the Dielectric Properties of Solid Solutions Li_x_Ca_(1−x)_Cu_3_Ti_4_O_12_ (x = 0.01–0.1). J. Compos. Sci..

[28] Havriliak S., Negami S. (1967). A Complex Plane Representation of Electronical and Mechanical Relaxation. Polymer.

[29] Melo B., Loureiro F.J., Fagg D.P., Costa L., Graça M. (2021). DFRTtoEIS An Easy Approach to Verify the Consistency of a DFRT Generated from an Impedance Spectrum. Electrochim. Acta.

[30] Maglione M., Vikhnin S., Liu Springer G.K. (2010). Polarons, Free Charge Localisation and Effective Dielectric Permittivity in Oxides. arXiv.

[31] Deskins N.A., Dupuis M. (2007). Electron Transport via Polaron Hopping in Bulk TiO_2_: A Density Functional Theory Characterization. Phys. Rev. B Condens. Matter Mater. Phys..

[32] Yagi E., Hasiguti R.R., Aono M. (1996). Electronic Conduction above 4 K of Slightly Reduced Oxygen-Deficient Rutile TiO_2−x_. Phys. Rev. B Condens. Matter.

